# Effect of salicylic acid pretreatment on the postharvest response of hardy kiwifruit during storage

**DOI:** 10.1080/15592324.2025.2572018

**Published:** 2025-10-19

**Authors:** Uk Lee, Hyun Ji Eo, Chung Ryul Jung, Yonghyun Kim

**Affiliations:** Special Forest Resources Division, National Institute of Forest Science, Gwonseon-gu, Suwon, Republic of Korea

**Keywords:** Hardy kiwifruit, salicylic acid, ripening, ethylene, antioxidant

## Abstract

Hardy kiwifruit (*Actinidia arguta*) is a climacteric fruit, a characteristic contributing to its short shelf life. Plant phytohormones such as salicylic acid (SA) are well known for their role in regulating the postharvest fruit ripening processes. Here, we investigated, for the first time, the effect of SA pretreatment on postharvest responses in the hardy kiwifruit cultivar ‘Autumn Sense’ during cold storage. SA pretreatment effectively maintained fruit firmness and titratable acidity during the first two weeks of storage, whereas both parameters declined sharply in untreated control fruits. Moreover, no ethylene production was detected in SA-pretreated fruits during the same period, likely due to modulation of gene expression in the ethylene biosynthetic pathway. These results suggest that SA pretreatment suppresses the early phases of ripening, thereby delaying fruit softening in hardy kiwifruit during cold storage. In addition, antioxidant activity and ascorbic acid content were significantly upregulated in fruits treated with 0.1 mM SA during the first week, indicating enhanced antioxidant accumulation. Overall, these findings provide valuable insights into the postharvest physiology of hardy kiwifruit and support the use of SA pretreatment as a strategy to extend shelf life and improve fruit quality in commercial storage and distribution.

## 1. Introduction

*Actinidia arguta*, commonly known as minikiwi, baby kiwi, or hardy kiwifruit, is cultivated worldwide in various countries, including New Zealand, Australia, the United States, Chile, South Korea, China, and many European nations.^1^ The fruit is notable for its compact size and nutrient density, containing exceptionally high levels of vitamin C, phenolic compounds, potassium, and lutein.^1^^,^^2^ These attributes not only contribute to its health-promoting properties—including antioxidant, anti-inflammatory, and hypoglycemic effects—but also enhance its appeal as a functional food crop in the nutraceutical market.^2^^,^^3^ Hardy kiwifruit is typically harvested at a physiologically immature stage, approximately 100–130 days after full bloom, although this period varies slightly among cultivars.^4^ After harvest, ripening is stimulated by the high respiration rate and ethylene production of the fruit,^5^^,^^6^ both of which are essential for developing the desirable taste and texture expected by consumers. However, the climacteric nature of hardy kiwifruit represents a critical barrier to effective postharvest management.^7^ As the fruit continues to soften during the ripening process, it becomes increasingly susceptible to mechanical damage during transportation and distribution. Consequently, extending the shelf life and maintaining the quality of hardy kiwifruit throughout the supply chain remain major challenges faced by the commercial market.

Salicylic acid (SA), a naturally occurring phenolic compound, has gained substantial interest as a multifunctional plant hormone involved in growth regulation, defense signaling, and abiotic stress tolerance.^8^^,^^9^ SA, which is endogenously synthesized via the isochorismate and phenylalanine ammonia-lyase pathways, plays a critical role in systemic acquired resistance, inducing the expression of pathogenesis-related (PR) proteins and enhancing antioxidant defense systems.^10^^,^^11^ Exogenous application of SA has been shown to activate reactive oxygen species (ROS)-scavenging enzymes such as superoxide dismutase, catalase, and ascorbate peroxidase, which collectively enhance plant resilience to drought, salinity, heat, cold, and heavy metal stress.^12^^,^^13^ In fruit crops, SA has demonstrated additional benefits, including improved fruit firmness, delayed senescence, and enhanced storage stability. Studies conducted on strawberries, grapes, bananas, and citrus fruit have shown that both preharvest and postharvest SA treatments can significantly improve biochemical quality attributes and extend shelf life by modulating ethylene production and antioxidant response.^14^^,^^15^ In kiwifruit species, SA plays a specific role in the ripening process.^16^ Exogenous application of acetyl-SA, a chemical derivative of SA, has been shown to inhibit ethylene production by interfering with its biosynthesis and perception pathways, thereby delaying fruit ripening during storage.^17^ Nevertheless, empirical data on the physiological and biochemical effects of SA in *A. arguta* remain scarce.

Given the short shelf life of hardy kiwifruit due to its climacteric nature, and the proven efficacy of SA in extending the storage life of other fruit crops, further investigation into the effects of exogenous SA on the storage quality of hardy kiwifruit is warranted. Therefore, this study aimed to evaluate the impact of exogenous application of SA on fruit ripening and biochemical quality of *A. arguta* during cold storage. The findings are expected to contribute to sustainable postharvest management practices and enhance the resilience and marketability of this high-value fruit.

## 2. Materials and methods

### 2.1. Plant material and SA pretreatment

Hardy kiwifruits (*Actinidia arguta* ‘Autumn Sense’) were harvested at the commercial maturity stage, determined by soluble solids content (approximately 10–12%) and firmness (approximately 6–7 Newton), 110 to 115 days after full bloom, prior to full ripening on the vine. The fruits were collected from five-year-old trees grown in an orchard located in Wonju, Gangwon-do, Republic of Korea. Undamaged and uniform fruits were selected and divided into four groups for subsequent treatments [control, distilled water (DW), 0.1 mM SA, and 1 mM SA], with each group containing approximately 7 kg of fruit (300–340 fruits). For SA pretreatment, fruits were soaked for 5 min in either DW, 0.1 mM SA, or 1 mM SA solutions (SA; Sigma-Aldrich, St. Louis, MO, USA), then air-dried thoroughly on mesh trays for 2 hours. Non-treated fruits were used as the control. After drying, 22–25 fruits (approximately 500–520 g) were packed into perforated polyethylene terephthalate (PET) boxes. A total of 14 of these PET boxes per treatment were placed into plastic containers, which were then stored under controlled conditions at 3 °C with relative humidity maintained at 85–90% throughout the storage period.

### 2.2. Measurement of quality attributes

Fruit weight loss was monitored throughout the storage period using an electronic scale. Weights were recorded for each PET box at designated time points in triplicate, and weight loss was expressed as a percentage relative to the initial weight at the start of storage. Fruit firmness was measured using a texture analyzer (CT3; AMETEK Brookfield, Middleboro, MA, USA) fitted with a 2 mm diameter flat probe with 8–10 biological replicates. The center of the fruit's flat surface was penetrated to a depth of 10 mm with the probe at a constant speed of 1 mm/s. Firmness was recorded in Newtons (*N*). Total soluble solids content (TSSC) was determined using a digital refractometer (PR-101a; ATAGO, Tokyo, Japan) with 8–10 biological replicates and expressed as a percentage. Titratable acidity (TA) was measured with a Titrator EasyPlus Easy pH system (Mettler Toledo, Columbus, OH, USA) with 10 biological replicates, and results were reported as the percentage of citric acid equivalents.

### 2.3. Sample preparation for biochemical analyses

At each designated time point, sampled hardy kiwifruits, including both peel and pulp, were lyophilized using a freeze dryer (MG-VFD20; MG Industry, Gunpo, South Korea). The dried samples were then ground into a fine powder using a laboratory grinder (IKA Multidrive Basic; IKA Korea, Seoul, South Korea). The resulting powdered material was used for subsequent biochemical analyses.

### 2.4. Measurement of starch and soluble sugar content

Starch and soluble sugars were extracted from 0.1 g of fine powder of ten freeze-dried fruits per replicate, following the method described by Eo et al.^18^ The concentrations of starch and soluble sugars were determined in triplicate using enzymatic assay kits specific to starch and sucrose/D-glucose/D-fructose, respectively (R-Biopharm AG, Darmstadt, Germany), according to the manufacturer's instructions.

### 2.5. Determination of respiration rate and ethylene production of hardy kiwifruit

To measure respiration rate and ethylene production, three hardy kiwifruits were weighed and placed into 270 mL sealed containers fitted with a septum per replicate. After a 4-hour incubation at either 25 ± 1 °C prior to storage or 3 °C during storage, 1 mL of headspace gas was withdrawn through the septum. Carbon dioxide concentration was measured using a headspace gas analyzer (CheckMate 3; Mocon Dansensor Inc., Ringsted, Denmark) in triplicate, and ethylene concentration was determined in triplicate using a gas chromatography (GC) system (7890B; Agilent Technologies, Santa Clara, CA, USA). The GC system was equipped with a flame ionization detector (FID) and a PoraPLOT Q column (25 m × 0.32 mm; Agilent Technologies). Operating conditions were as follows: injector temperature, 150 °C; column temperature, 80 °C; detector temperature, 280 °C. Helium was used as the carrier gas at a flow rate of 4 mL/min. Ethylene production was expressed in microliters per kilogram per hour (µL·kg⁻¹·h⁻¹).

### 2.6. Total RNA isolation

Total RNA was isolated from hardy kiwifruit using the RNeasy Plant Mini Kit (Qiagen, Hilden, Germany), with minor modifications to the manufacturer's protocol. Frozen fruit samples stored at −80 °C were ground into a fine powder in liquid nitrogen using a mortar and pestle. Approximately 100 mg of this powder was lysed in 450 μL of RLT buffer supplemented with 3% (v/v) *β*-mercaptoethanol. The lysate was homogenized using a QIAshredder spin column (Qiagen) by centrifugation at 20,000 × *g* for 2 min. The flow-through was combined with 225 μL of 99.5% ethanol and transferred to an RNeasy spin column. RNA binding was achieved by centrifugation at 10,000 × *g* for 1 min. The column was then washed with 350 μL of RW1 buffer under the same centrifugation conditions. To remove genomic DNA, 80 μL of RNase-Free DNase (Qiagen) was applied to the column and incubated at room temperature for 15 min. Subsequently, the column was washed with 350 μL of RW1 buffer and twice with 500 μL of RPE buffer, with centrifugation at 10,000 × *g* for 1 min and 2 min, respectively. To remove residual ethanol, the column was placed in a new collection tube and centrifuged at 15,000 × *g* for 1 min. Finally, total RNA was eluted by adding 30 μL of nuclease-free water to the membrane, followed by centrifugation at 10,000 × *g* for 1 min. The eluted RNA was stored at −80 °C for further gene expression analysis.

### 2.7. Gene expression analysis

Reverse transcription-quantitative polymerase chain reaction (RT-qPCR) was employed to analyze gene expression in hardy kiwifruit with 3–4 biological replicates. A total of 250 ng of purified total RNA was used for cDNA synthesis using the PrimeScript RT reagent kit with gDNA Eraser (Takara Bio, Shiga, Japan), following the manufacturer's protocol. RT-qPCR was performed on a CFX96 Touch Real-Time PCR Detection System (Bio-Rad, Hercules, CA, USA) using iQ™ SYBR® Green Supermix (Bio-Rad) in a final reaction volume of 20 μL, including 2 μL of cDNA template. Relative expression levels of target genes were calculated using the comparative CT (ΔΔCT) method, as described by Schmittgen and Livak.^19^ The *AaActin* gene was used as an internal reference for normalization.^20^ Primer sequences used for gene amplification were obtained from Lin et al.^20^ and Lim et al.,^21^ and are listed in Table S1 in the Supplementary Material.

### 2.8. Determination of the total chlorophyll, carotenoid, flavonoid, phenolic, and ascorbic acid content

The extraction and quantification of total chlorophylls, carotenoids, flavonoids, phenolics, and ascorbic acid (AsA) were carried out in triplicate using extracts prepared from fine powder of ten lyophilized fruits per replicate, following the method previously described by Eo et al.^18^

### 2.9. Measurement of antioxidant activity

Antioxidants were extracted from 0.2 g of fine powder of ten lyophilized fruits per replicate following the method described by Eo et al.^18^ The 2,2-diphenyl−1-picrylhydrazyl (DPPH) radical scavenging activity was evaluated based on the method described by Stoilova et al.,^22^ with slight modifications. In brief, 0.2 mL of the extract was mixed with 1 mL of 0.4 mM DPPH solution and incubated in the dark at 25 °C for 30 minutes. Absorbance was then measured at 517 nm using a spectrophotometer (Epoch 2; Agilent Technologies, Santa Clara, CA, USA) in triplicate, and results were expressed as the percentage of DPPH scavenging activity. The 2,2'-Azino-bis (3-ethylbenzothiazoline−6-sulfonic acid (ABTS)-radical scavenging activity was measured according to the method of Biglari et al.,^23^ with minor modifications. A 7 mM ABTS in 2.45 mM potassium persulfate was prepared and incubated in the dark at 25 °C for 16 h. The resulting ABTS stock solution was diluted with 70% ethanol to an absorbance of approximately 1.5 at 734 nm to obtain the working solution. Following this, 30 μL of the extract was added to 1 mL of the diluted ABTS solution, and the mixture was incubated at 25 °C for 6 minutes. Absorbance was immediately measured at 734 nm using a spectrophotometer (Epoch 2) in triplicate, and ABTS scavenging activity was calculated and expressed as a percentage.

### 2.10. Statistical analysis

All data are presented as mean ± standard deviation (SD). Statistical significance between treatments at each time point was assessed using one-way ANOVA followed by Tukey's Honestly Significant Difference (HSD) test (*p* < 0.05). All statistical analyses were performed with GraphPad Prism version 10 (GraphPad Software, San Diego, CA, USA).

## 3. Results

### 3.1. Effect of SA pretreatment on the quality attributes of hardy kiwifruit during cold storage

**Figure 1.**
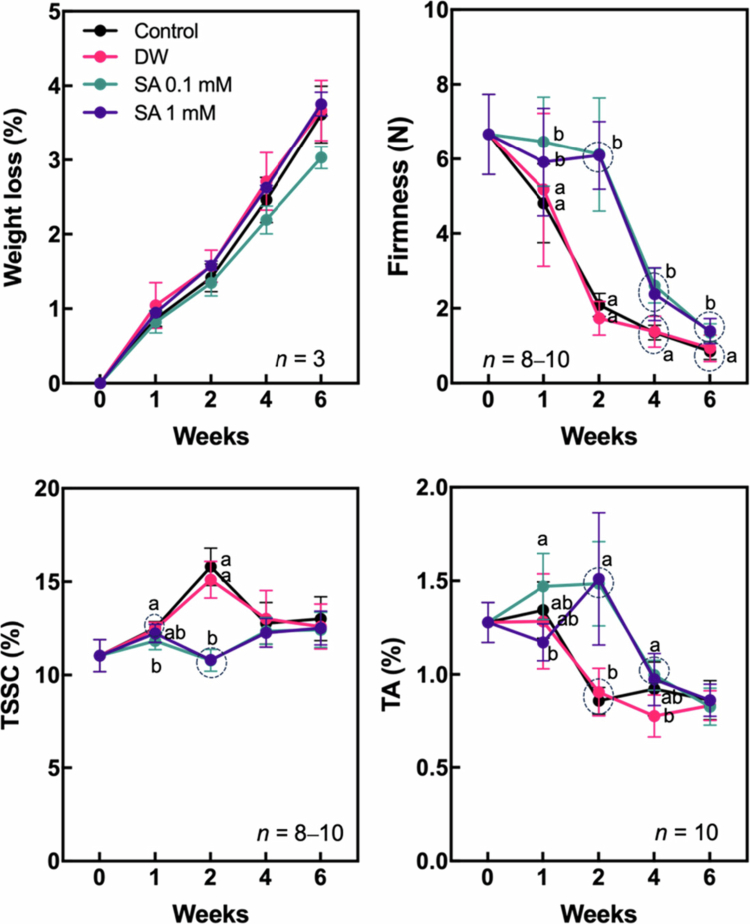
Changes in physicochemical properties of hardy kiwifruit during storage. Hardy kiwifruit was pretreated with salicylic acid (SA) prior to storage. Measured parameters included weight loss, firmness, total soluble solids content (TSSC), and titratable acidity (TA). Different letters indicate statistically significant differences between treatments, determined using one-way ANOVA followed by Tukey's Honestly Significant Difference (HSD) test at *p* < 0.05.

Hardy kiwifruit undergoes gradual ripening after harvest during cold storage. This ripening process is marked by an increase in TSSC and a decrease in fruit weight, TA, and firmness.^18^^,^^24^ Fruit weight loss steadily increased throughout the entire storage period across all treatments (Figure 1). Although the differences between control and SA pretreatments were not statistically significant, the 0.1 mM SA treatment exhibited a slightly lower trend in weight loss compared to the others. Notably, fruits pretreated with 0.1 mM and 1 mM SA maintained their firmness for up to two weeks, while fruits in the control and DW treatments showed a sharp decline in firmness over the same period. Although firmness decreased noticeably in the SA-treated fruits after two weeks, the differences between the control and SA groups remained apparent up to six weeks. TSSC increased rapidly in the control and DW groups during the first two weeks of storage. In contrast, fruits treated with 0.1 mM and 1 mM SA maintained TSSC levels similar to those at the beginning of storage. From four weeks, TSSC values had leveled off and showed no significant differences between control and SA pretreatments. TA of control and DW groups declined steeply during the first two weeks of cold storage and remained relatively constant thereafter. In SA-treated fruits, however, TA was better maintained during the initial two weeks, followed by a notable reduction from four weeks. Physiological disorders such as shriveling, browning, pitting, and decay were monitored during cold storage, with no significant differences observed among treatments. However, peel browning scores were slightly higher in control fruits compared to those pretreated with SA (Figure S1 in the supplementary material).

**Figure 2.**
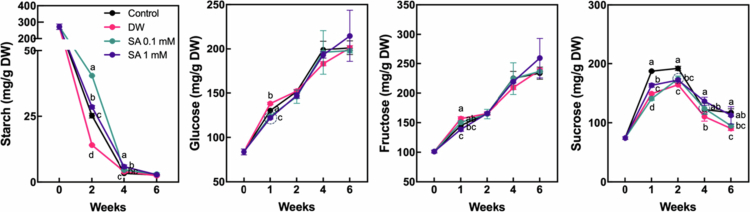
Changes in starch and soluble sugar content in hardy kiwifruits during storage. Hardy kiwifruit was pretreated with salicylic acid (SA) prior to storage. Different letters indicate statistically significant differences between treatments, determined using one-way ANOVA followed by Tukey's Honestly Significant Difference (HSD) test (*p* < 0.05, *n* = 3).

Starch degradation and the accumulation of soluble sugars are associated with fruit softening in hardy kiwifruit,^25^ indicating that these metabolic processes are actively triggered during ripening. To evaluate the effects of SA pretreatment on carbohydrate metabolism related to fruit ripening, starch and soluble sugar levels were measured throughout the cold storage period. Starch levels in fruits pretreated with 0.1 mM SA declined more slowly than in control and the other treatments, resulting in significantly higher starch content after two weeks (Figure 2). Additionally, the SA-treated group maintained significantly higher starch levels than the control at four weeks, although the values were substantially lower compared to those observed at two weeks. Conversely, glucose and fructose levels increased consistently in all treatments during the storage period, with significant differences among treatments detected only at the one-week mark. Sucrose content increased in all treatments during the first two weeks of storage, followed by a decline up to six weeks. Notably, the control group exhibited a significantly higher sucrose level during the initial two weeks compared to the other treatments. Taken together, these results suggest that SA pretreatment delayed fruit ripening partially during at least the first two weeks of storage in spite of the ongoing decline in fruit weight and the accumulation of soluble sugars. This highlights the potential of SA to help preserve fruit freshness by delaying the ripening process during the early phases of cold storage.

### 3.2. The effects of SA pretreatment on the respiration and ethylene production

**Figure 3.**
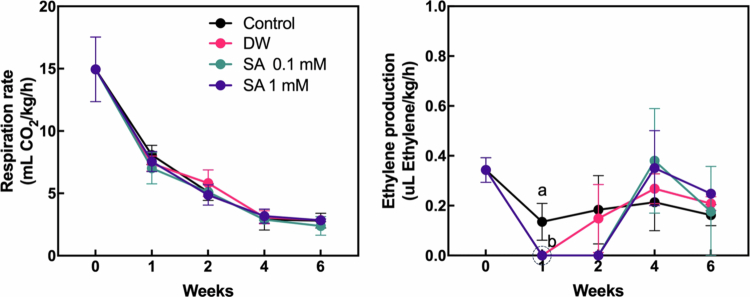
Respiration rate and ethylene production of hardy kiwifruit during storage. Hardy kiwifruit was pretreated with salicylic acid (SA) prior to storage. Different letters indicate statistically significant differences between treatments, determined by one-way ANOVA followed by Tukey's Honestly Significant Difference (HSD) test (*p* < 0.05, *n* = 3).

**Figure 4.**
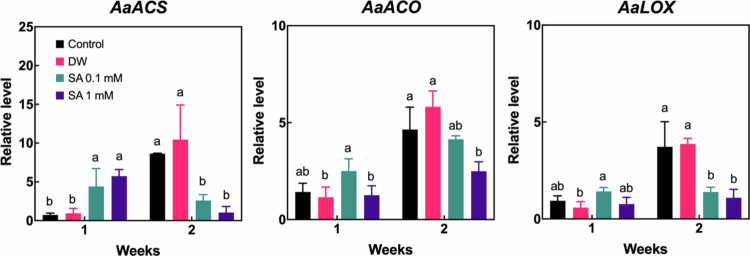
Relative transcript levels of *1-aminocyclopropane−1-carboxylate synthase (AaACS)*, *1-aminocyclopropane−1-acid carboxylic oxidase (AaACO)*, and *lipoxygenase* (*AaLOX)* analyzed by qPCR. Gene expression was analyzed in hardy kiwifruit two weeks after storage following salicylic acid (SA) pretreatment. Different letters indicate statistically significant differences between treatments, determined using one-way ANOVA followed by Tukey's Honestly Significant Difference (HSD) test (*p* < 0.05, *n* = 3–4).

Fruit respiration and ethylene production are critical processes regulating ripening in climacteric fruits.^26^ As hardy kiwifruit exhibits climacteric behavior, we investigated the effects of SA pretreatment on fruit respiration rate and ethylene production to evaluate its influence on the ripening process (Figure 3). The respiration rate of the fruit gradually declined throughout the cold storage period, and no significant differences were observed between the control and SA-treated groups. However, no ethylene production was detected in SA-pretreated fruits during the first two weeks of storage, whereas ethylene was consistently detected in the control group throughout the entire storage period. To further examine the molecular effect of SA pretreatment on ethylene production and ripening process, we analyzed the expression of genes related to ethylene biosynthesis and ripening—*1-aminocyclopropane−1-carboxylic acid synthase* (*AaACS*), *1-aminocyclopropane−1-carboxylic acid oxidase* (*AaACO*) and *lipoxygenase* (*AaLOX*)^21^^,^^27^—during the initial two weeks of storage (Figure 4). At one week, *AaACS* expression was upregulated in both 0.1 mM and 1 mM SA-treated fruits compared to the control. However, after two weeks, the control fruits exhibited markedly higher *AaACS* expression than the SA-pretreated groups. Similarly, *AaACO* expression was significantly lower in fruits treated with 1 mM SA compared to the control at the two-week mark. In addition, both 0.1 mM and 1 mM SA pretreatments appeared to maintain relatively stable *AaLOX* expression levels during the first two weeks, whereas *AaLOX* expression was significantly upregulated in the control group at two weeks. These findings suggest that SA pretreatment suppresses ethylene production in hardy kiwifruit during the early phases of cold storage by inhibiting the increase in the expression of genes involved in ethylene biosynthesis, thereby delaying fruit ripening compared to untreated control fruits.

### 3.3. Changes in the level of antioxidant compounds by SA pretreatment

**Figure 5.**
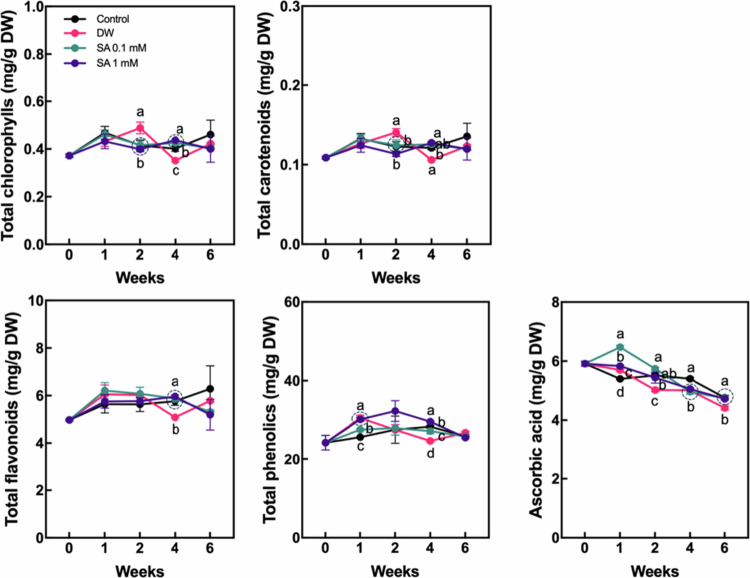
Changes in total chlorophyll, carotenoid, flavonoid, phenolic, and ascorbic acid levels in hardy kiwifruit during storage. Hardy kiwifruit was pretreated with salicylic acid (SA) prior to storage. Different letters indicate statistically significant differences between treatments, determined using one-way ANOVA followed by Tukey's Honestly Significant Difference (HSD) test (*p* < 0.05, *n* = 3).

**Figure 6.**
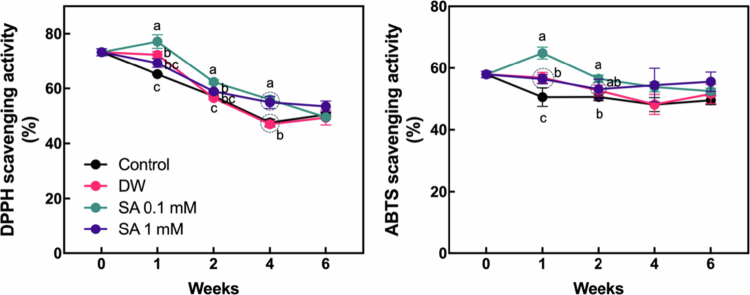
Changes in antioxidant activity of hardy kiwifruit during storage. The hardy kiwifruit was pretreated with salicylic acid (SA) prior to storage. Different letters indicate statistically significant differences between treatments, determined using one-way ANOVA followed by Tukey's Honestly Significant Difference (HSD) test (*p* < 0.05, *n* = 3).

SA plays a key role as a signaling molecule in regulating ROS production and indirectly modulates the level of secondary metabolites, such as antioxidants, in plants.^12^^,^^28^ To evaluate the effect of SA pretreatment on antioxidant accumulation in hardy kiwifruit during cold storage, we measured the levels of total chlorophylls, total carotenoids, total flavonoids, total phenolics, and AsA throughout the storage period (Figure 5). The levels of total chlorophylls and total carotenoids were generally maintained throughout the storage period across all treatments. Although slight differences were observed at two and four weeks after the start of storage, SA-pretreated fruits did not exhibit substantial differences compared to the control. Total flavonoid levels showed a slight increase after the start of storage across all treatments, in which SA-pretreated groups did not exhibit a significant difference in flavonoid concentrations compared to the control fruits. In contrast, total phenolic content was markedly higher in fruits pretreated with 1 mM SA for up to six weeks compared to the control, whereas fruits pretreated with 0.1 mM SA showed phenolic levels comparable to those of the control group. Notably, AsA levels decreased throughout the storage period across all treatments following the onset of storage. However, a significant increase in AsA content compared to the control was observed only in fruits pretreated with 0.1 mM SA at one week. These results suggest that the levels of certain antioxidant components in hardy kiwifruit are influenced by SA pretreatment, with the effects varying according to the concentration of SA applied. To compare the overall antioxidant potential between the control and SA-pretreated groups, DPPH and ABTS assays were performed using fruit samples (Figure 6). Fruits pretreated with 0.1 mM SA exhibited significantly higher antioxidant activity than both the control and 1 mM SA-treated fruits during the first week, as measured by both DPPH and ABTS assays. This significant difference in antioxidant activity was maintained for up to four or six weeks compared to the control in DPPH and ABTS assays, respectively, suggesting that 0.1 mM SA pretreatment enhances antioxidant potential during the early phases of storage and contributes to improved antioxidant capacity throughout the storage period.

**Figure 7.**
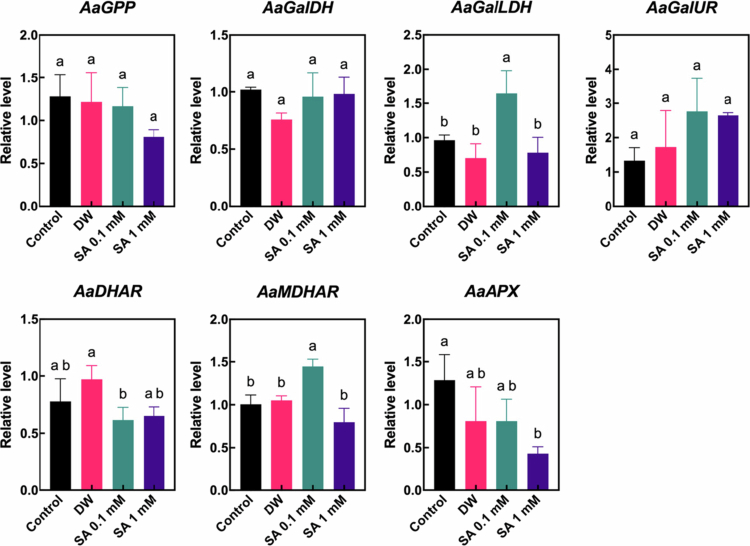
A relative transcript levels of ascorbic acid biosynthesis [*L-galactose dehydrogenase* (*AaGalDH*)*, L-galactono−1,4-lactone dehydrogenase* (*AaGalLDH*)*, L-galactose−1-phosphate phosphatase* (*AaGPP*), and *D-galacturonate reductase* (*AaGalUR*)] and regeneration [*dehydroascorbate reductase* (*AaDHAR*)*, monodehydroascorbate reductase* (*AaMDHAR*), and *ascorbate peroxidase* (*AaAPX*)] pathways related genes in hardy kiwifruit analyzed by qPCR. Gene expression was analyzed in hardy kiwifruit at one week of storage following salicylic acid (SA) pretreatment. Different letters indicate statistically significant differences between treatments, determined using one-way ANOVA followed by Tukey's Honestly Significant Difference (HSD) test (*p* < 0.05, *n* = 3–4).

AsA is a major antioxidant in hardy kiwifruit, which contains a high amount of AsA compared to other *Actinidia* species.^29^^,^^30^ To better understand the molecular response of SA-induced AsA biosynthesis and regeneration pathways, we investigated the expression level of several genes involved in AsA biosynthesis and regeneration pathways using fruit samples collected one week after the start of storage, due to the level of AsA only being upregulated at the time point by SA (Figure 7). The expression levels of *L-galactose−1-phosphate phosphatase* (*AaGPP*), *L-galactose dehydrogenase* (*AaGalDH*), and *L-galactono−1,4-lactone dehydrogenase* (*AaGalLDH*)—key genes in the L-galactose pathway, the predominant route for AsA biosynthesis—were examined. Among these, only *AaGalLDH* was significantly upregulated by the 0.1 mM SA treatment. *D-galacturonate reductase* (*AaGalUR*), a gene involved in an alternative AsA biosynthesis pathway, was upregulated following both 0.1 mM and 1 mM SA pretreatments; however, the differences were not statistically significant. In the AsA regeneration pathway, the expression of the gene encoding ascorbate peroxidase (AaAPX), which catalyzes the conversion of H₂O₂ into water with AsA serving as the electron donor,^31^ was suppressed in SA-pretreated fruits, with a significant downregulation observed in the 1 mM SA treatment. The expression of the gene encoding dehydroascorbate reductase (AaDHAR), which regenerates AsA from DHA,^31^ was not notably affected by SA pretreatment. In contrast, the expression of the gene encoding monodehydroascorbate reductase (AaMDHAR), which regenerates AsA from MDHA,^31^ was significantly upregulated in fruits treated with 0.1 mM SA. The findings indicate that SA pretreatment partially stimulates the molecular mechanisms of AsA biosynthesis and regeneration, leading to AsA accumulation in hardy kiwifruit during the early phase of cold storage.

## 4. Discussion

### 4.1. Relationship between exogenous SA and hardy kiwifruit ripening

Postharvest ripening is an essential process for hardy kiwifruit, as the fruit is typically harvested at an immature stage and is not ready to eat for consumers at the time of harvest. However, ripening also presents a challenge for prolonged storage and distribution, as the softening that occurs during this process makes the fruit more susceptible to mechanical damage.^6^^,^^7^ Therefore, regulating the ripening process plays a crucial role in extending the shelf life and preserving the high quality and market value of the hardy kiwifruit. To extend the shelf life of hardy kiwifruit by delaying the ripening process, various approaches have been widely studied to date. Low-temperature storage is a widely used and well-established method for preserving hardy kiwifruit. However, prolonged or suboptimal cold storage can lead to chilling injuries such as peel pitting, browning, and shrinkage, with the severity of damage varying depending on the storage temperature and fruit cultivar.^18^^,^^32^ Short-term anaerobic treatment has been shown to delay ripening and softening in hardy kiwifruit by reducing ethylene production and starch degradation, as well as simultaneously activating the antioxidant system.^33^ The edible alginate-based coating method has been shown to enhance the storability of hardy kiwifruit during cold storage.^34^ Krupa et al.^35^ reported that treatment with 1-methylcyclopropene (1-MCP) combined with low-oxygen conditions effectively reduced loss of firmness and delayed the increase in TSSC during the storage period. SA is recognized as an endogenous plant growth regulator involved in various physiological processes, including the enhancement of biotic and abiotic stress tolerance. It has also shown considerable potential in delaying fruit ripening, improving fruit quality, and reducing postharvest losses in both climacteric and non-climacteric fruits.^36^ The role of exogenous SA in modulating ripening processes, especially fruit softening, has been established in multiple fruit cultivars, including fuzzy kiwifruit.^37^ In particular, treatment with acetyl-SA has been shown to increase endogenous SA levels in kiwifruit. This increase is positively correlated with enhanced fruit firmness and reduced ethylene production, suggesting that endogenous SA levels may be associated with maintaining fruit firmness.^16^ However, phytohormones such as SA have not yet been extensively applied in the postharvest treatment of hardy kiwifruit. In the present study, we investigated the effect of SA pretreatment on the ripening process of hardy kiwifruit during cold storage. SA-pretreated fruits effectively maintained fruit firmness and exhibited reduced starch degradation during the early phase of storage compared to control fruits (Figures 1 and 2). Additionally, ethylene production was suppressed during the same period (Figure 3). These findings are consistent with results reported in previous studies on other members of the *Actinidia* genus,^14^^,^^37^ suggesting that the effectiveness of SA treatment in hardy kiwifruit may be attributed to genetic traits shared with other species within the *Actinidia* genus. Similarly, the expression levels of *AaACS* and *AaACO* did not gradually increase in SA-pretreated fruits during the early phase of cold storage, although *AaACS* was upregulated at the first week (Figure 4). Previously, it was demonstrated that SA inhibits ethylene formation from its precursor, 1-aminocyclopropane−1-carboxylic acid, in plants.^38^ Recent studies have reported that most members of the *AdACS* and *AdACO* gene families in fuzzy kiwifruit are downregulated by exogenous acetyl-SA treatment, whereas *AdACS3* and *AdACO3* are specifically upregulated.^39^ In this case, the activation of AdACS3 and AdACO3 is regulated by AdMPK16 (a mitogen-activated protein kinase) and AdAP (an aspartic peptidase), respectively. Both transcripts encoding these proteins are downregulated by exogenous acetyl-SA, suggesting that ethylene biosynthesis is partially regulated at the post-transcriptional level. While the precise molecular mechanisms by which SA inhibits ethylene biosynthesis in hardy kiwifruit are yet to be fully clarified, previous studies partially support the notion that SA treatment may regulate the ripening process in hardy kiwifruit cultivars by modulating ethylene production at both transcriptional and post-transcriptional levels during the postharvest period. Furthermore, in the present study, the effect of SA did not extend into the late phase of storage with respect to ethylene production, which was observed after four weeks in SA-pretreated fruits. This finding suggests that the inhibitory effect of SA on ethylene production may diminish during the late phase of storage. Although it remains unclear whether the pretreated SA was degraded or converted into an inactive form, further investigation of SA metabolism in the fruit after pretreatment could provide a deeper understanding of its precise role in regulating ethylene production during the late phase of storage. Moreover, in this study, hardy kiwifruit were soaked in an SA solution prior to cold storage, a treatment that could not be applied continuously. Unlike SA, methyl salicylate (MeSA), a volatile derivative of SA, can be applied continuously during fruit storage and has been shown to significantly influence the fruit ripening process.^15^ Although the impact of MeSA on hardy kiwifruit requires further investigation, it may represent a promising alternative for delaying ripening and maintaining fruit freshness throughout the storage period.

### 4.2. SA modulated fruit antioxidants at the postharvest stage

Hardy kiwifruit is regarded as one of the most nutrient-rich fruits, providing a valuable source of health-promoting compounds.^2^ Hardy kiwifruit cultivars contain substantial levels of chlorophylls, carotenoids, and polyphenols, with the levels of chlorophylls and polyphenols being more than twice as high as those in *A. deliciosa*.^29^ Moreover, some hardy kiwifruit cultivars contain considerably higher levels of AsA compared to *A. chinensis* and *A. deliciosa.*^29^^,^^40^ In the present study, we characterized the effects of SA pretreatment on the levels of health-promoting compounds in hardy kiwifruit. In particular, the levels of AsA and phenolics varied during the early phase and the entire storage period, respectively, following SA pretreatment, depending on the SA concentration (Figure 5). In addition, nonspecific antioxidant activities, such as DPPH and ABTS radical scavenging activities in hardy kiwifruit, were also upregulated by 0.1 mM SA pretreatment during the early phase of storage. In particular, the pattern of changes in their levels during storage was similar to that observed for AsA following SA pretreatment, suggesting that AsA may be a major contributor to the antioxidant potential of hardy kiwifruit. Because SA is known to be a signaling molecule and plays a major role in activating antioxidant systems in various crop plants,^13^^,^^15^ the upregulation of certain antioxidant levels and activity in the early phase of storage appears to be a reasonable response to exogenous SA in hardy kiwifruit. In particular, as shown in Figure 7, several genes related to AsA biosynthesis and regeneration were partially up- or downregulated in response to exogenous SA, depending on the SA concentration, during the early phase of cold storage. This suggests that SA-induced gene expression changes contribute to the increased levels of AsA. Furthermore, it can be speculated that the differential responses of antioxidant groups to exogenous SA reflect their distinct roles in postharvest physiological processes during cold storage. Previous studies have shown that reactive oxygen species (ROS) are closely associated with fruit ripening events, such as softening and coloration; however, excessive ROS accumulation can have detrimental effects on fruit quality throughout the postharvest period.^41^ In this context, SA-induced antioxidants, such as AsA, may help mitigate ROS levels, thereby partially contributing to delayed postharvest ripening in hardy kiwifruit under cold storage conditions. Further research aimed at elucidating the specific roles of individual antioxidants in the ripening process would offer valuable insights into the regulation of postharvest physiology in hardy kiwifruit.

## 5. Conclusion

Regulating the ripening process of hardy kiwifruit is a critical factor in improving its storability and marketability, as the fruit is prone to softening as ripening progresses. Various physical and chemical approaches have been examined to prolong the shelf life of hardy kiwifruit to date.^32^ Here, we demonstrated that SA pretreatment improved fruit firmness and suppressed ethylene production by modulating the ethylene biosynthetic pathway during the early phase of cold storage. Additionally, SA pretreatment regulated the levels of specific antioxidants, such as phenolics and AsA. These findings indicate that exogenous SA acts as an effective modulator of the ripening process and the accumulation of health-promoting compounds in hardy kiwifruit during cold storage. Although large-scale application trials are needed to verify the practical efficacy of SA pretreatment at the industrial storage level and to assess cultivar-specific responses, SA pretreatment is a promising and effective alternative tool for prolonging the shelf life and enhancing the intrinsic quality of hardy kiwifruit.

## Supplementary Material

Supplementary material **Figure S1.** The effect of SA pretreatment on the incidence of physiological disorders in hardy kiwifruit during storage. Shriveling, browning, pitting, and decay were assessed using a 6-point scale: 0 = 0%, 1 = 1–20%, 2 = 21–40%, 3 = 41–60%, 4 = 61–80%, and 5 = 81–100% of the affected area on the fruit surface (n = 18–20).**Table S1.** List of primers used in RT-qPCR.

## Data Availability

The data that support the findings of this study are available from the corresponding author upon reasonable request.
