# Supplementary material for: Effect of salicylic acid pretreatment on the postharvest response of hardy kiwifruit during storage
Source: Plant Signal Behav. 2025 Oct 19;20(1):2572018. doi: 10.1080/15592324.2025.2572018 (PMC12536623; doi:10.1080/15592324.2025.2572018)
Supplement: Supplementary material — Figure S1. The effect of SA pretreatment on the incidence of physiological disorders in hardy kiwifruit during storage. Shriveling, browning, pitting, and decay were assessed using a 6-point scale: 0 = 0%, 1 = 1–20%, 2 = 21–40%, 3 = 41–60%, 4 = 61–80%, and 5 = 81–100% of the affected area on the fruit surface (n = 18–20).Table S1. List of primers used in RT-qPCR. [file KPSB_A_2572018_SM8364.docx]

**Supplementary material**

**Title**: Effect of salicylic acid pretreatment on the postharvest response of hardy kiwifruit during storage

**Author information:**

**Authors**

Uk Lee^1^, Hyun Ji Eo^1^, Chung Ryul Jung^1^, and Yonghyun Kim^1^*

**Author affiliations**

^1^Special Forest Resources Division, National Institute of Forest Science, Gwonseon-gu, Suwon 16631, Republic of Korea

***Corresponding author**

yonghyun24@korea.kr

**
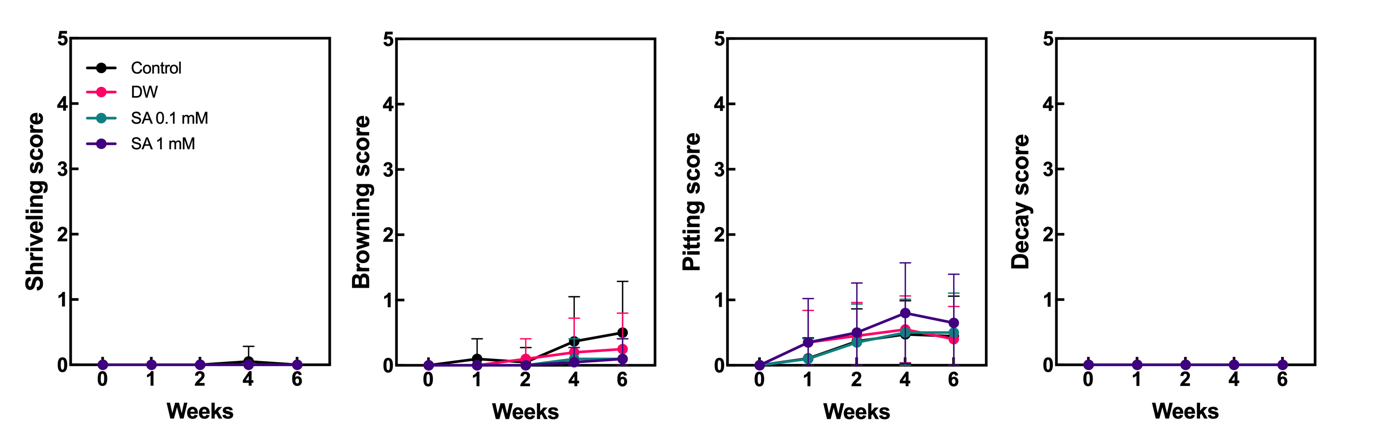
**

**Figure S1.** The effect of SA pretreatment on the incidence of physiological disorders in hardy kiwifruit during storage. Shriveling, browning, pitting, and decay were assessed using a 6-point scale: 0 = 0%, 1 = 1–20%, 2 = 21–40%, 3 = 41–60%, 4 = 61–80%, and 5 = 81–100% of the affected area on the fruit surface (*n* = 18–20).

**Measurement of physiological disorders**

The incidence of physiological disorders such as shrinking, browning, pitting, and decay of the fruit was assessed in 18–20 biological replicates using a 6-point scale based on the percentage of the fruit surface affected by each symptom: 0 = 0%, 1 = 1–20%, 2 = 21–40%, 3 = 41–60%, 4 = 61–80%, and 5 = 81–100% of the affected area.

**Table S1.** List of primers used in RT-qPCR

| Gene |  | Primer Sequence (5'-3') |
| --- | --- | --- |
| *AaACS* | Forward | CAACCTCCTGCTCACGTTCA |
| (Lim et al., 2016) | Reverse | GTTGGAGTATATGGCCCCGA |
| *AaACO* | Forward | TGCTTGTGAGAACTGGGGCTT |
| (Lim et al., 2016) | Reverse | GCGCAAGAAGAAGGTGCTTTC |
| *AaLOX* | Forward | CAGCCGGGAGTGCCTGCTCTG |
| (Lim et al., 2016) | Reverse | CATGCAGTAATCGAGCCATTC |
| *AaGPP* | Forward | TGTGACGATTGCTGATAA |
| (Lin et al., 2022) | Reverse | ACCATTCTCCTCTCCATA |
| *AaGalDH* | Forward | GATTGTGAATGAGACGATT |
| (Lin et al., 2022) | Reverse | GGAGTCCTGTAATACCAA |
| *AaGalLDH* | Forward | TCCTATTGACGAGCAAGT |
| (Lin et al., 2022) | Reverse | AGCGAGCAAGATAGAAGA |
| *AaGalUR* | Forward | GATGCCGGACCTGGAAACT |
| (Lin et al., 2022) | Reverse | ACTTGATGATGCCGAGATGG |
| *AaDHAR* | Forward | GCCGCTTCTGGTTCTCCTGAT |
| (Lin et al., 2022) | Reverse | TCGGAGTCAGGAATCCATTTGTC |
| *AaMDHAR* | Forward | TAGAAGCAGACACGATTG |
| (Lin et al., 2022) | Reverse | ACCATCAACCTGTATTCC |
| *AaAPX* | Forward | TTGTTCCGTCCATATTGA |
| (Lin et al., 2022) | Reverse | TTATTCTCTGGTCTGCTAAT |
| *AaActin* | Forward | GCTTACAGAGGCACCACTCAACC |
| (Lin et al., 2022) | Reverse | CCGGAATCCAGCACAATACCAG |

**References**

Lim et al., 2016, Inhibition of hardy kiwifruit (*Actinidia arguta*) ripening by 1-methylcyclopropene during cold storage and anticancer properties of the fruit extract. Food Chem. 190, 150-157.

Lin et al., 2022, L-ascorbic acid metabolism in two contracting hardy kiwifruit (*Actinidia arguta*) cultivars during fruit development, Sci. hortic. 297:110940
